# Psychiatry residents’ perceptions of competence acquisition, training programe compliance and clinical supervision in the Spanish psychiatry training system

**DOI:** 10.1192/j.eurpsy.2023.2382

**Published:** 2023-07-19

**Authors:** J. Esteve Aznar, J. P. Carrasco Picazo, J. I. Etxeandía Pradera, E. J. Aguilar García-Iturrospe

**Affiliations:** 1 Psychiatry, Hospital Clínico Universitario de Valencia; 2 Psychiatry, INCLIVA, Valencia; 3 Psychiatry, CIBERSAM, Madrid; 4 Psychiatry, Universidad de Valencia, Valencia, Spain

## Abstract

**Introduction:**

There are differences in the training curricula of medical specialists in different countries. The opinion of the doctors in training on how they acquire competencies and carry them out is of great importance. In our case, we asked ourselves what were the perceived shortcomings in psychiatric training.

**Objectives:**

The main objective of the study is to describe the opinion of psychiatry residents in Spain on the acquisition of competencies, compliance with the training programme and quality of clinical supervision.

**Methods:**

This is a descriptive, cross-sectional, mixed (quantitative and qualitative) study. Based on previous bibliography and the ministerial order of the official training programme, an online survey was prepared, which was disseminated telematically through the residents’ representatives of the National Commission of the Speciality of Psychiatry of the National Council of Health Sciences Specialities.

**Results:**

A total of 109 responses were obtained, with representation from all the Autonomous Communities of Spain. Graph I shows the opinion of the psychiatry residents as to which competencies they feel are less developed at present, with the competencies related to psychotherapy standing out in first place with great importance. In terms of compliance with the training programme, the parameter most in line with what was established was the average number of shifts, with an average of 4.26 shifts per month. However, 11.7% of residents do not take compensatory rest after on-call duty as required by law. Moreover, the rotation times established by the BOE are not complied with in 38.5% of the hospitals. With regard to the rotations that the residents feel should increase their rotation time, the child and adolescent psychiatry and dual pathology rotations stand out (graph II). Finally, with regard to the supervision process, only 22.90% of first-year residents are always supervised in person during their rotations (graph III).

**Image:**

**Figure:**
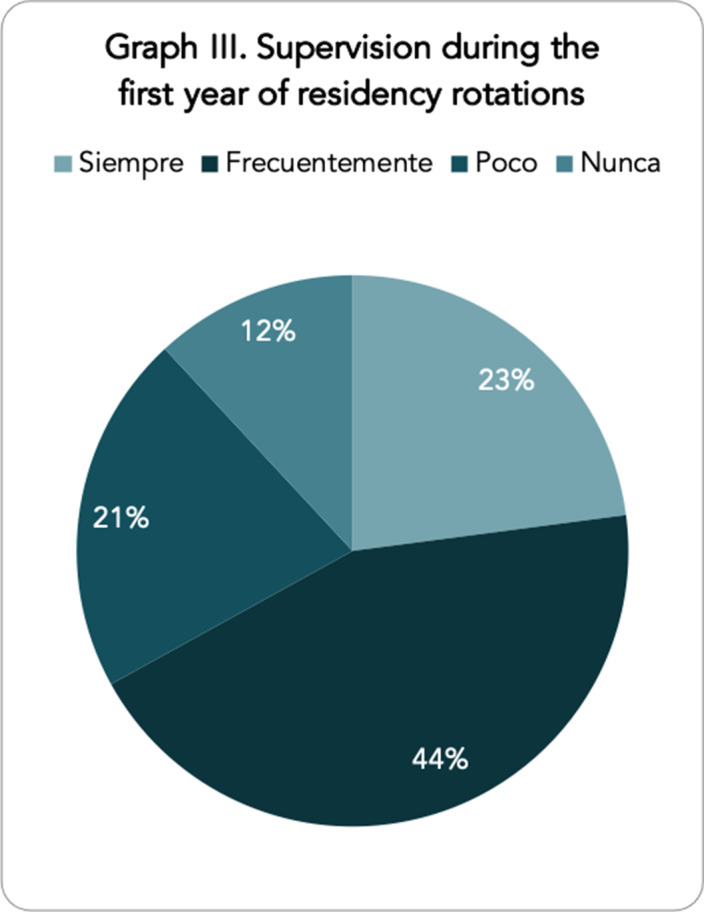

**Image 2:**

**Figure:**
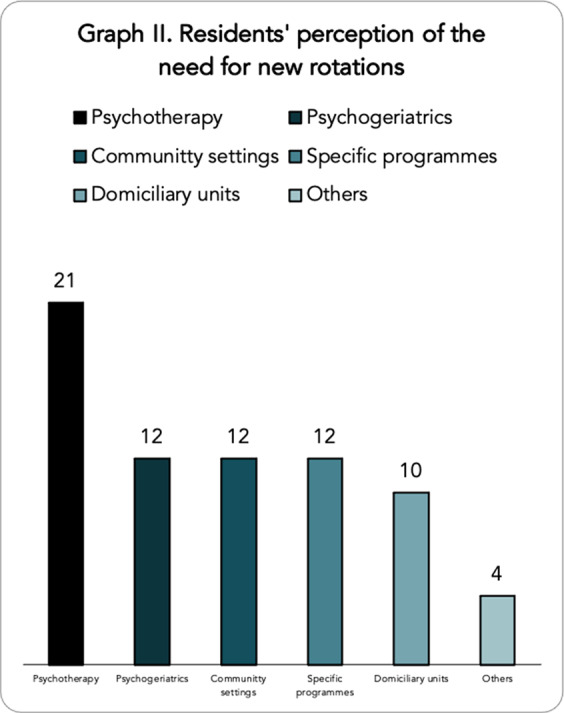

**Image 3:**

**Figure:**
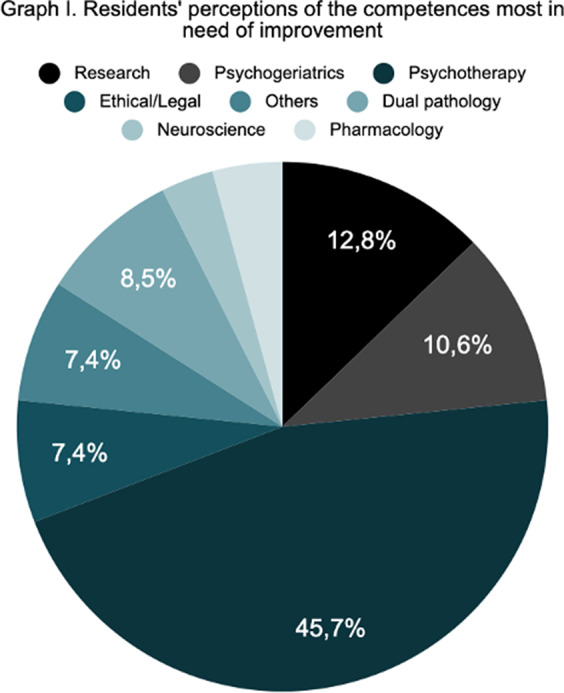

**Conclusions:**

Our study reflects the belief on the part of the resident physicians that further training in psychotherapy (45.7%), psychogeriatrics (10.6%) and dual pathology (8.5%) would be necessary. The fact that only 11.7% of the participants stated that they did not take compensatory rest after on-call duty seems to us to be an improvement over what was initially expected. There are other less reassuring data, such as the fact that only 22.9% of first-year residents report having continuous supervision. We consider that the results found follow the trends observed in studies carried out in residents from other countries. We stress the need to carry out a greater number of studies with a broad population base in which to find the failures that psychiatry residents themselves perceive in their training.

**Disclosure of Interest:**

None Declared

